# Emotional Modulation of Response Inhibition in Adolescents During Acute Suicidal Crisis: Event-Related Potentials in an Emotional Go/NoGo Task

**DOI:** 10.1177/15500594211063311

**Published:** 2021-12-13

**Authors:** Meggan Porteous, Paniz Tavakoli, Kenneth Campbell, Allyson Dale, Addo Boafo, Rebecca Robillard

**Affiliations:** 1 Sleep Research Unit, 151181 University of Ottawa, Institute of Mental Health Research at the Royal, Ottawa, ON, Canada; 2 School of Psychology, 6363 University of Ottawa, Ottawa, ON, Canada; 3 ARiEAL Research Centre, McMaster University, Hamilton, ON, Canada; 4 274065 Mental Health Research Unit, 27338 Children's Hospital of Eastern Ontario, Ottawa, ON, Canada; 5 6363 Department of Psychiatry, University of Ottawa, Ottawa, ON, Canada

**Keywords:** inhibition, emotion, suicidality, adolescence, ERPs

## Abstract

*Objectives.* Suicide is the second leading cause of adolescent deaths and may be linked to difficulties with inhibitory and emotional processing. This study assessed the neural correlates of cognitive inhibition during emotional processing in adolescents hospitalized for a suicidal crisis. *Methods.* Event-related potentials were recorded during an emotional Go/NoGo task in 12 adolescents who attempted suicide and 12 age- and sex-matched healthy controls. *Results.* Compared to the control group, the suicidal group showed significantly reduced positivity at the time of the P3d (difference waveform reflecting NoGo minus Go trials) in response to happy and neutral, but not sad stimuli. For happy stimuli, this group difference was restricted to the right hemisphere. Further analyses indicated that the suicidal group had a reversed pattern of P3 amplitude in response to inhibition, with lower amplitudes in the NoGo compared to the Go conditions. Suicidal symptoms severity strongly correlated with lower amplitude of the P3d in response to neutral faces. *Conclusions.* These findings provide more insight into inhibition difficulties in adolescents with acute suicidal risk. Interactions between emotional and inhibition processing should be considered when treating acutely suicidal youths.

## Introduction

Worldwide, a suicide attempt is made every 3 s, and an attempt is completed every 40 s.^1^ Suicide is the second leading cause of death in adolescents worldwide.^2^ Nearly 14% of Canadian adolescents report serious suicidal ideation.^3^ Among the many factors influencing adolescent suicide, difficulties with inhibitory control have been consistently observed and may contribute to increased risk of future suicidal behaviour.^4–6^

In adults, difficulties with inhibitory control have been proposed to increase susceptibility to suicidality by altering one's ability to inhibit intrusive suicidal thoughts.^7,8^ This may be more difficult in the context of emotion processing, whereby the cognitive resources needed to inhibit the urge to enact suicidal thoughts may be altered by a negative emotional state. Inhibitory processes broadly operate through the active suppression (or inhibition) of inappropriate responses to irrelevant distractor stimuli.^9^ This has been investigated, for instance, with the Stop-Signal task, in which one must suppress a preplanned response when prompted by a “stop” signal. On this task, individuals engaging in self-harm have been shown to commit more errors than nonsuicidal individuals, specifically in response to negative emotional images as opposed to neutral or positive images.^10^ These findings suggest a link between self-harm and poor inhibitory control in the context of emotional processing.

Adolescence is a critical time for brain maturation, especially for regions involved in inhibitory control and emotional regulation.^11–13^ Notably, top-down modulation of voluntary behavior may not operate at maximum efficiency in adolescence compared to adulthood.^14–16^ Emotion processing and inhibitory control have both been found to improve from adolescence to adulthood, and emotionally salient information has been shown to negatively impact inhibitory control.^14,17^ As a result, suicidality and inhibitory control may interact differently in adolescents than in adults.

To date, little research has focused on inhibitory control in adolescent suicidality. Behavioral impulsivity studies have reported no differences in response inhibition.^18–20^ However, there are indications that people who attempted suicide have greater impulsivity than healthy controls on self-report measures and respond more impulsively to reward-based tasks.^18–20^

### Inhibitory Control and Emotional Processing

Emotional processing is often altered in suicidal individuals.^21–25^ For instance, suicidal ideators have an attentional bias towards negatively valenced emotional stimuli related to suicide.^21,25^ It has been argued that suicidal ideation may in fact result from impaired emotional regulation rather than intense negative emotions per se.^25^ Difficulties in inhibitory control can also negatively impact emotional regulation strategies, such as reappraisal and distraction, further impairing one's ability to manage difficult emotions.^26–28^ More specifically, inhibition has been found to be a key component in accessing mood-incongruent stimuli in an effort to mediate negative mood states.^27^ Interactions between cognitive inhibition processes and emotional processing may thus play a critical role in modulating negative mood-states and increase the vulnerability for suicide.

### Go/NoGo Paradigm

One of the most widely used paradigms for the study of response inhibition is the Go/NoGo task.^29^ During this task, participants are instructed to respond upon presentation of an equiprobable “Go” stimulus but to withhold this response following presentation of a “NoGo” stimulus, whereby the “Go” trials prime the behavioral response. The processing of Go stimuli is thought to reflect activation of decision-making and memory-updating processes, while NoGo stimuli reflect response inhibition processes. Inhibitory control is composed of 2 underlying neural processes: identifying a need for inhibition (ie, conflict detection)^29,30^ and actively inhibiting the prepared response (ie, motor inhibition).^31,32^ Because participants are asked not to respond on NoGo trials, the extent of processing leading to the inhibition of the response is difficult to determine using strict performance measures.

Many studies have recorded event-related potentials (ERPs) to measure the differential processing of Go and NoGo stimuli (for reviews, see^33,34^), particularly in the absence of an overt response. Accurate detection of Go stimuli is associated with 2 main ERP components: Go-N2, occurring at about 200 to 250 ms, is thought to reflect the extent of cognitive effort required for the controlled detection of the stimulus event, and Go-P3, occurring at about 350 to 450 ms, may reflect memory-updating and active attentional-decisional processes. The NoGo stimuli are associated with a NoGo-N2 perhaps reflecting conflict monitoring or novelty detection, followed by the NoGo-P3, which is thought to reflect the actual inhibition processing and/or the conscious decision to withhold a response.^34^

Previous studies revealed abnormal brain responses to Go/NoGo tasks in adults with depression. For instance, Ruchsow et al (2008) measured ERPs in a NoGo-Go difference waveform in patients with depression and healthy controls. This subtraction removes processing that is common to both stimulus events, leaving processing that is unique to the NoGo condition (ie, inhibition). Their results indicated similar N2d deflection (ie, component corresponding to N2 in the difference waveform) in both groups. However, compared to the control group, the depression group had a smaller P3d (ie, lower difference between the Go and NoGo conditions at the time of the P3). This was related to a reduced P3 amplitude in the NoGo condition and suggests fewer resources available for inhibition processes in adults with depression. Few studies on suicidality have employed ERP methods. A recent Go/NoGo study in adults reported that individuals with a history of suicide had a larger increase in N2 amplitude from Go to NoGo trials compared to adults with suicidal ideation who had no history of attempts.^35^ Hence, adults who attempted suicide may require greater cognitive resources to detect conflict when the inhibition of a response is required.

Go/NoGo paradigms using emotionally charged stimuli may be especially relevant to disorders characterized by an abnormal sensitivity to affective stimuli. In adolescents with major depressive disorder, the amplitude of the NoGo-N2 was smaller to positive compared to negative stimuli.^36^ This might reflect a need for increased effort and difficulty in recruiting executive resources to inhibit a response to positive stimuli. A similar study using the Stop-Signal paradigm in adults with depression reported that the Stop-N2 was reduced in response to positive images compared to neutral images, suggesting lower neural resources for conflict detection involving emotional images.^37^ They also noted that the amplitude of the Stop-P3 was reduced for both positive and negative images compared to neutral images, suggesting that high emotional valence may lead to altered inhibitory processing.

To our knowledge, no study has examined the neural correlates of cognitive inhibition and emotional processing in adolescents with suicidal behavior. The present study aimed to identify potential differences in the neural mechanisms underlying inhibitory control in the context of emotional processing between suicidal and nonsuicidal adolescents. Considering the putative preponderant implication of abnormalities affecting the right hemisphere for depression and suicidality^38^, we also aimed to assess hemispheric differences.

## Methods

### Participants

Participants were drawn from the sample of a previous study.^39^ The participants were 12 (9 females) adolescents admitted to the inpatient psychiatric unit of a pediatric hospital because of an acute risk of suicide and 12 age- and sex-matched healthy controls. Participants ranged in age from 13 to 17 years (mean = 14.8, SD = 1.39 years) and all were right-handed. All participants from the suicidal group had a diagnosis of major depression as assessed based on criteria from the Diagnostic and Statistical Manual of Mental Disorders, Fifth Edition (DSM-V), reported a plan to kill themselves with the full intention of dying, and had made a suicide attempt. At the time of admission, suicidal risk was judged to be too high for these adolescents to safely be in the community. All participants from the suicidal group were taking psychotropic medication; 11 out of 12 participants were taking antidepressants (selective serotonin reuptake inhibitors; SSRIs), and smaller proportions were taking atypical antipsychotics, mood stabilizers/anticonvulsants, melatonin, or stimulants (please see Table 1). For the suicidal group, clinical interviews with the participants and their families were conducted by a board-certified psychiatrist from the clinical team within 24 h of their admission to determine diagnosis based on DSM-V criteria. Individuals with schizophrenia, neurological, or pervasive developmental disorders were systematically excluded. A brief screening interview was conducted with the control participants to ensure that they had no prior diagnosis of any mental health conditions or history of admission to a mental health facility. None of the participants were taking benzodiazepine medication or had reported hearing or neurological disorders. Written informed consent was obtained from all participants, and parents when the participant was younger than 16 years of age, prior to the start of the study. The study was approved by both the University of Ottawa's Health Sciences and Science Research Ethics Board and the Children's Hospital of Eastern Ontario Research Institute's Research Ethics Board (CHEOREB# 13/155X). The study was conducted according to the Declaration of Helsinki guidelines on ethical conduct involving human subjects.

**Table 1.** Demographic and Clinical Characteristics of the Control and Patient Groups.

	Control (n = 12)	Suicidal (n = 12)
Age: mean (SD)	14.42 (1.2)	15.08 (1.5)
Sex distribution (n [% females])	12 (66.7)	12 (75.0)
Medication intake (n [%])		
Antidepressants	0 (0%)	11 (91.7%)
Mood stabilizers/anticonvulsants	0 (0%)	1 (8.3%)
Melatonin	0 (0%)	2 (16.7%)
Stimulants	0 (0%)	2 (16.7%)
Atypical antipsychotics	0 (0%)	4 (33.3%)
CDI-2: mean (SD)		
Total score	7.8 (7.5)	25.4 (7.6)
Emotional problems	4 (3.9)	13 (4.3)
Negative mood and physical symptoms	2.5 (2.1)	8 (3.0)
Negative self-esteem	1.5 (2.0)	6.9 (3.1)
Functional problems	3.6 (3.8)	12.8 (4.3)
Ineffectiveness	2.9 (2.3)	9.1 (3.7)
Interpersonal problems	0.8 (1.7)	3.9 (1.6)
SBQ: mean (SD)	4.9 (2.7)	15 (2.3)

Note. CDI, Children's Depression Inventory second Edition; SBQ, Suicidal Behavior Questionnaire; SD, Standard Deviation.

### Psychological Assessment

The severity of depressive symptoms was assessed using the Children's Depression Inventory-2 (CDI-2)^40^ a commonly used self-report rating inventory that includes 27 items. The 27 items are grouped into 2 major factors, each comprised of 2 subscales assessing emotional problems (including negative mood/physical symptoms and negative self-esteem) and functional problems (including ineffectiveness and interpersonal problems). The presence and severity of suicidal symptoms were assessed using the Suicidal Behaviors Questionnaire-Revised (SBQ-R),^41^ a brief 4-item, self-report questionnaire measuring different dimensions of suicidality: (1) lifetime suicide ideation and suicide attempt, (2) frequency of suicide ideation over the past 12 months, (3) threat of suicidal behavior, and (4) the likelihood of suicidal behavior.

### Procedure and Stimuli

Participants were engaged in an emotional version of a Go/NoGo task.^42^ The stimuli included images of emotional facial expressions (happy, sad, neutral) of 10 adults (5 males and 5 females) cropped to an elliptical shape to eliminate the hair and background cues. Participants sat ∼1 m in front of a computer monitor. Each face subtended a visual angle of ∼4° × 3°. Four blocks of stimuli were presented. In 2 of the blocks, equiprobable neutral and happy faces were presented in random order. In one of these blocks, participants were asked to press the space bar with the index finger of their dominant hand (Go trials) upon detection of the neutral face and to withhold the response (NoGo trials) upon detection of the happy face. In the other block, the response procedure was reversed (ie, Go to the happy face, NoGo to the neutral face). In the remaining 2 blocks, equiprobable neutral and sad faces were presented again in random order. Again, the facial expression that required a response (Go trials) and that which did not (NoGo trials) were reversed in the 2 blocks. The same stimuli were used in Go and NoGo conditions.

Each block contained 124 trials. The order of the 4 blocks was randomized. Each stimulus within a block was presented for 500 ms and the interstimulus interval was 2000 ms. The stimulus presentation procedure and recording of responses was controlled by the E-Prime software (Psychology Software Tools, Pittsburgh, USA).

### Electrophysiological Recording

Electroencephalography (EEG) was recorded using BrainAmp amplifiers and Recorder software (Brain Products, Gilching, Germany). The EEG was recorded from 13 electrodes (F3, Fz, F4, C3, Cz, C4, P3, Pz, P4, O1, O2, M1, M2) according to the 10/20 system of electrode placement. The nose served as a reference for all channels. Vertical eye movements and blinks (vEOG) were recorded from electrodes placed at the supraorbital ridge of the eye. Horizontal eye movements (hEOG) were recorded from electrodes placed on the outer canthus of each eye. Interelectrode impedances were kept below 5 kΩ. The high-frequency filter was set at 75 Hz and the time constant was set at 2 s. The electrical signals were digitized continuously using 500 Hz sampling rate.

### ERP Analysis

The data were reconstructed using Brain Products’ Analyzer2 software. The continuous EEG data was band-pass filtered at 0.5 to 20 Hz (24 dB/octave). A vertical EOG channel was computed by subtracting activity recorded at supraorbital and infraorbital ridges of the left eye. A horizontal EOG channel was computed by subtracting activity recorded at the outer canthus of each eye. Independent Component Analysis^43^ was used to identify eye movement and blink artifacts that were statistically independent of the EEG activity. These were then partialled out of the EEG traces. The continuous data were subsequently reconstructed into discrete single trial 900 ms segments, beginning 100 ms before stimulus onset and then baseline corrected. Segments in which EEG activity exceeded  ± 100 μV relative to the baseline were excluded from further analyses. No more than 5% of total trials were rejected from further analyses per participant. The single trials were then sorted and averaged based on stimulus type (Go or NoGo), emotional facial expression (happy, sad, neutral) and electrode site.

### Quantification Analyses

Amplitudes of all ERP components where measured relative to the mean zero-voltage prestimulus baseline. They were quantified for each individual participant using the mean of all the data points within ± 25 ms of the peak amplitude that was identified in the grand average (the average of all participants' averages) separately for the control group and suicidal groups.

Visual inspection of the grand average raw data revealed N2 and P3 components to both Go and NoGo stimuli. The N2 was identified as the most negative peak between 150 and 300 ms after stimulus presentation. The P3 was identified as the most positive peak between 300 and 500 ms. The N2 was identified at the Fz electrode site and the P3 at the Pz electrode site, where they tend to be maximum in amplitude, based on visual inspection of the ERP waveforms.

**Figure 1.**
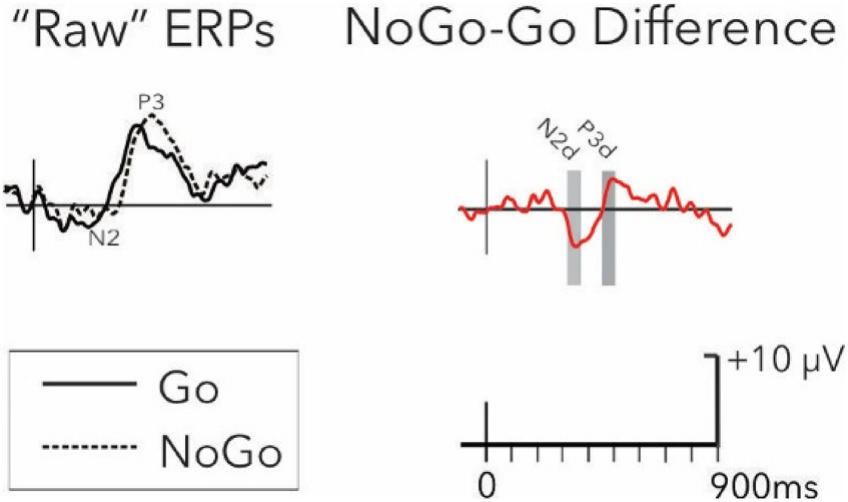
Difference wave calculations. Example of “raw” ERPs for Go (full line) and NoGo (dotted line) conditions (left panel), and example of difference waveform (NoGo minus Go; red line; right panel). The signal shown in this example was collected at the C4 electrode in the sad emotional condition in the control group.

A large portion of the processing involved in Go and NoGo trials is common (eg, initial sensory processing of the visual stimulus). To isolate the unique processing of the NoGo stimuli and examine the effects of inhibition, the Go waveforms were subtracted, point-by-point, from the NoGo waveforms.^44^ The subtraction procedure is illustrated in Figure 1. From these difference waveforms, the N2d was identified at Fz as the most negative-going peak between 150 and 350 ms after stimulus onset for each emotion and group separately. The P3d was identified at Pz as the most positive-going peak in the window of 300 to 500 ms poststimulus onset for each emotion and group separately.

It has been proposed that suicidality in adolescents may be linked to reduced EEG activation in the left hemisphere.^45^ Previous findings in related clinical samples (eg, depressed individuals) also suggest possible hemispheric differences in emotional face processing^46^ and Go/NoGo ERPs.^36^ Analyses were therefore focused on lateral electrodes. Previous work indicated that: (i) the difference between NoGo and Go trials on visual tasks are larger at central and frontal electrode sites,^45,47^ (ii) interactions between Go/NoGo conditions and emotional valence are larger at fronto-central sites,^48^ and (iii) interactions between Go/NoGo conditions and clinical groups (eg, depressed vs healthy controls) are more apparent at central than frontal and parietal electrodes.^49^ Hence, analyses were focused at central electrodes (C3 and C4).

### Statistical Analyses

Reaction times for Go trials were submitted to a two-way ANOVA with group (suicide attempters, controls) as a between-subject factor, and emotional (happy, neutral, sad) as a within-subject factor. Mean accuracy (percentage of correct trials) was submitted to a two-way ANOVA with group (suicide attempters, controls) as a between-subject factor, and with inhibition condition (Go, NoGo) and emotion (happy, neutral, sad) as within-subject factors.

The N2d and P3d ERP components measured in the difference waveforms were submitted to a three-way ANOVA with group (suicide attempters, controls) as a between-subject factor, and emotion (happy, neutral, sad) and hemispheres (left, right) as within-subject factors. When significant effects were found in difference waveforms, secondary analyses were run on the raw waveform components (N2 and P3) to confirm the direction of inhibition effects (four-way ANOVA with group [suicide attempters, controls] as a between-subject factor, and emotion [happy, neutral, sad], inhibition conditions [Go, NoGo], and hemispheres [left, right] as within-subject factors). Distribution normality was tested with the Shapiro-Wilk test.

Spearman correlations were conducted to determine how ERP components found to differ between groups correlated with the severity of depressive symptoms (CDI-2) and suicidal behaviors (SBQ-R) in all participants. A Bonferroni correction was applied to account for multiple testing.

Sensitivity analyses were conducted to determine whether significant results persisted: (a) without the single participant from the suicidal group who was not using antidepressant medications, and (b) after controlling for other types of psychotropic medications using ANCOVAs or partial correlations.

## Results

### Demographics

Demographic and clinical characteristics of both groups are listed in Table 1. The suicide attempters group had significantly higher scores compared to the controls on all subdomains of the CDI-2 and on the SBQs (*P* < .001).

### Performance Data

The overall mean RT on Go trials was 428 ms (SD = 51 ms). There was no significant group by emotion interaction or significant group difference for mean RTs. A main effect of emotion was found for mean RTs (*F*_[2, 44]_ = 12.2, *P *< .001, ηp2 = .36): RTs were faster with the happy faces (*M* = 414) compared to both the neutral (*M* = 430) and sad faces (*M* = 437; both *P* < .001).

There were relatively few errors of omissions (failing to respond to Go trials) and few errors of commission (failing to withhold the response on NoGo trials). Overall, mean accuracy across both groups was above 83% for all conditions. An interaction between inhibition condition and emotion (*F*_[2, 44]_ = 5.2, *P* = .009, ηp2 = .19) showed that accuracy was significantly lower for sad compared to both neutral (*P* < .001) and happy (*P* = .003) faces in NoGo trials, while there was no significant differences across emotional facial expressions in Go trials. There was no significant interaction involving group or significant group difference for accuracy.

### ERPs

**Figure 2.**
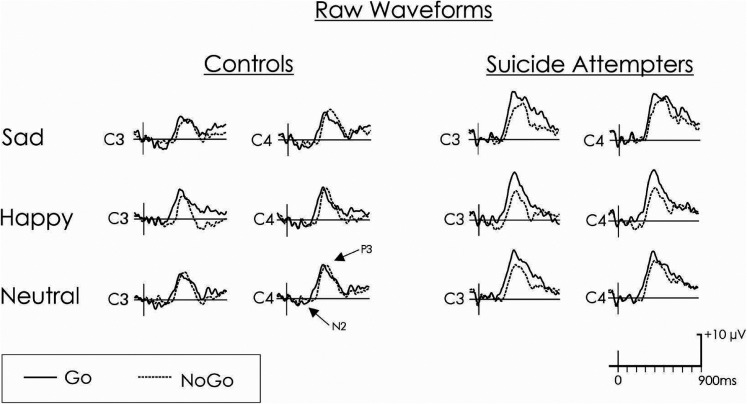
Raw waveforms. Grand average raw waveforms reflecting the NoGo (dotted line) and Go (black line) conditions for the control and suicidal groups at C3 (left hemisphere) and C4 (right hemisphere) electrode sites.

**Figure 3.**
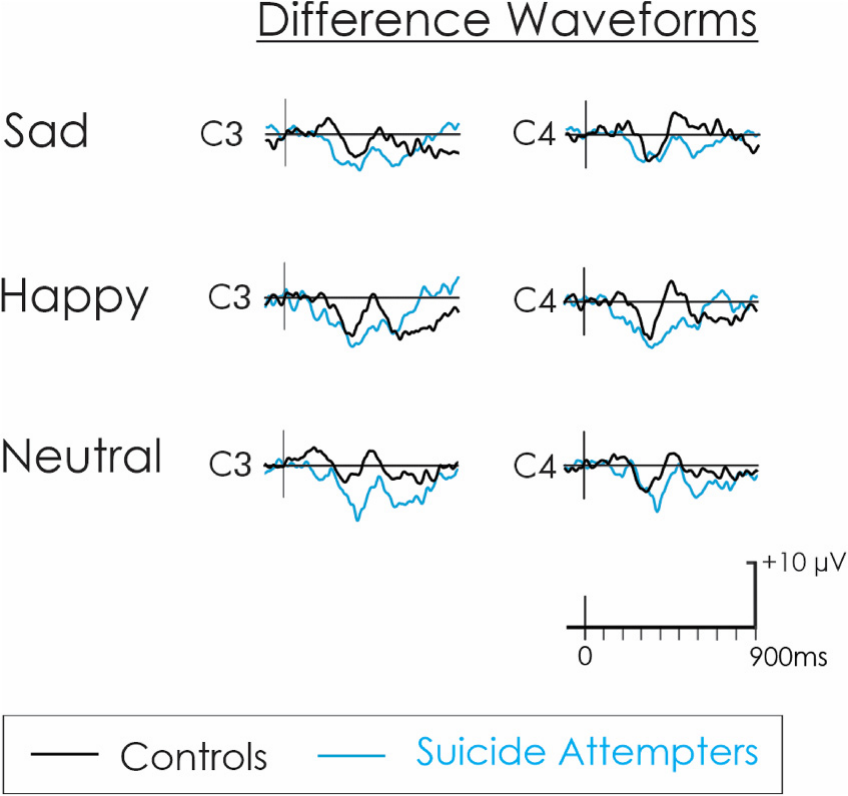
Difference waveforms. Grand average difference waveforms reflecting the NoGo minus Go trials for the control (black) and suicidal (blue) groups in C3 (left hemisphere) and C4 (right hemisphere) electrode sites.

ERPs for raw and difference waveforms (NoGo-Go trials) for the happy, neutral, and sad faces in the suicidal and control groups are shown in Figures 2 and 3, respectively.

#### Conflict Detection

A small N2 was elicited by the stimuli (Figure 2). The amplitude of the N2d was also small (Figure 3). There was no significant interaction or main effect of group, hemisphere, or emotion for N2d (all *P* > .050). There was no significant interaction or main effects for the latency of the N2d (*P* ≥ .050).

#### Inhibition

**Figure 4.**
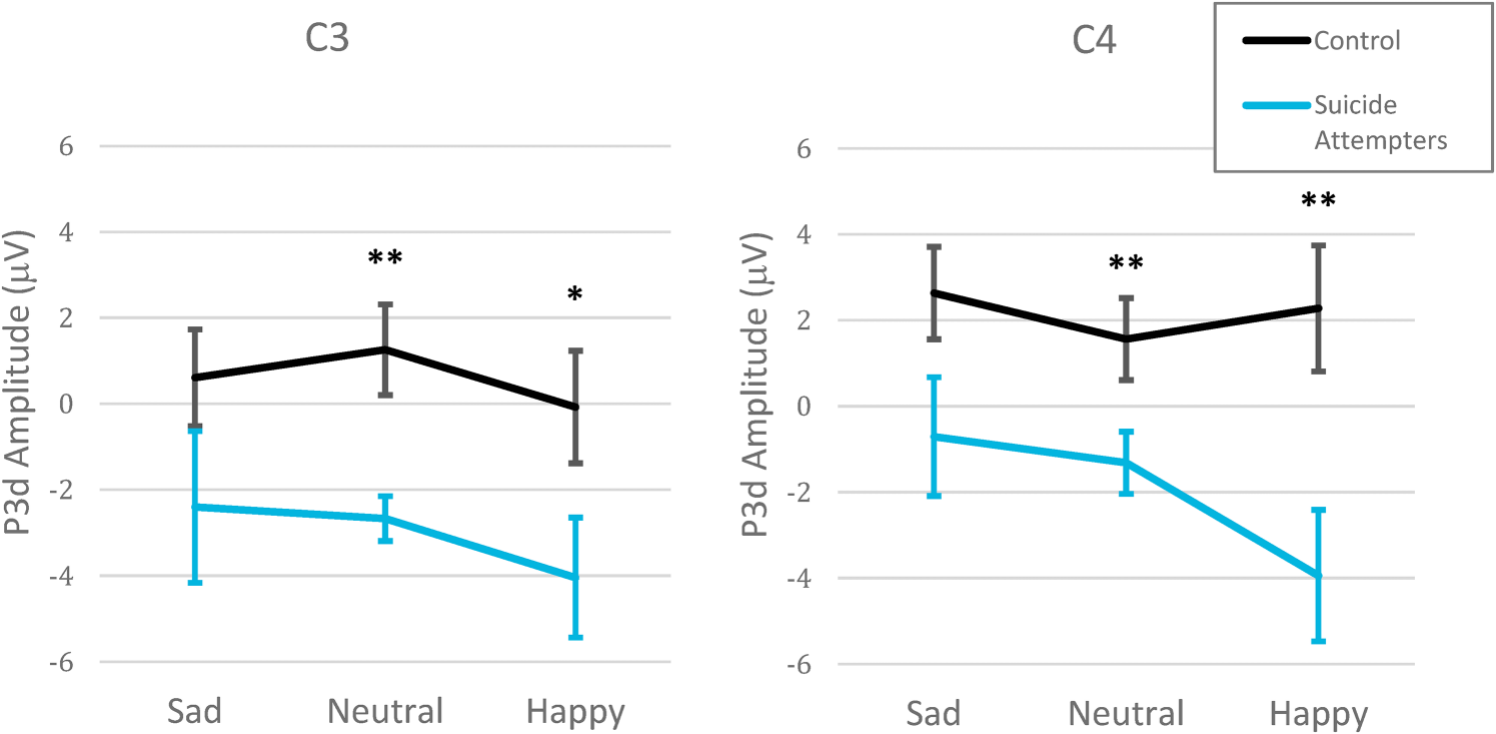
Group by inhibition conditions by emotional facial expression interaction for the P3d. Significant interaction between group (control vs suicidal), hemisphere (C3-Left vs C4-Right) and emotional valence (happy, neutral, sad) for the P3d (NoGo minus Go amplitudes) in central derivations. ** *P *< .010, * *P *< .050.

A large P3 was elicited by the stimuli (Figure 2), which yielded a clear P3d in the difference waveform (Figure 3). A significant three-way interaction between group, hemisphere and emotion was found for P3d (F_[2,44]_ = 3.7, *P* = .033, ηp2 = .14; Figures 3 and 4), a finding which remained significant without the single participant not taking antidepressant medication (*F*_[2,40]_ = 3.4, *P* = .044, ηp2 = .14) and after controlling for other psychotropic medications (*F*_[2,42]_ ≥ 3.5, *P *< .038, ηp2 > .14). This three-way interaction was linked to a significant group by hemisphere interaction emerging only with the happy faces (*F*_[1,22]_ = 10.8, *P* = .003, ηp2 = .33). Specifically, compared to the control group, the suicide attempters had a significantly reduced positivity at the time of the P3d in response to happy faces, and this was more pronounced over the right (C4: *P* = .008) compared to the left (C3: *P* = .049) hemisphere. A main group difference for emotionally neutral faces showed that, compared to the control group, the suicide attempters also had a significantly reduced positivity at the time of the P3d in response to neutral faces (*F*_[1,22]_ = 9.3, *P* = .006, ηp2 = .30). Specifically, for both happy and neutral faces, the control group had null or positive P3ds, and the suicide attempters had negative P3ds with larger absolute values (indicating that, in suicide attempters, the P3 decreased from the Go to the NoGo condition). For sad faces, there was no significant interaction or main group effect, but the overall P3d was significantly larger in the right (C4) compared to the left (C3) hemisphere (*F*_[1,22]_ = 10.1, *P* = .004, ηp2 = .31). There was no significant interaction or main effects for the latency of the P3d (*P* ≥ .050).

Secondary analyses on the raw P3 confirmed the direction of the inhibition effects suggested by the P3d difference waveform results. A significant group by inhibition condition interaction (*F*_[1,22]_ = 4.7, *P* = .041, ηp2 = .18) revealed similar P3 amplitudes in the Go and NoGo trials for the control group (*P* = .685), while in the suicide attempters, the P3 decreased significantly from the Go to the NoGo condition (*P* = .002). This group by inhibition condition interaction became a nonsignificant trend without the single participant not taking antidepressant medication (*F*_[2,42]_ = 3.4, *P* = .079, ηp2 = .14) and after controlling for stimulant medications (*F*_[2,42] _> 3.8, *P *< .066, ηp2 > .15), but remained after adjusting for mood stabilizers/anticonvulsants and atypical antipsychotics (F_[2,42]_ ≥ 4.8, *P *≤ .040, ηp2 > .19).

Furthermore, a significant inhibition condition by hemisphere interaction (*F*_[1,22]_ = 10.8, *P* = .003, ηp2 = .33) indicated that the decrease in the P3 from the Go to the NoGo condition was more pronounced in the left (C3: *P* = .002) than in the right (C4: *P* = .066) hemisphere. The inhibition condition by hemisphere interaction remained significant without the single participant not taking antidepressant medication (*F*_[2,42]_ = 10.6, *P* = .004, ηp2 = .33) and after controlling for other psychotropic medications (*F*_[2,42]_ > 7.0, *P *< .015, ηp2 > .25).

#### Associations Between ERPs and the Severity of Depressive and Suicidal Symptoms

**Figure 5.**
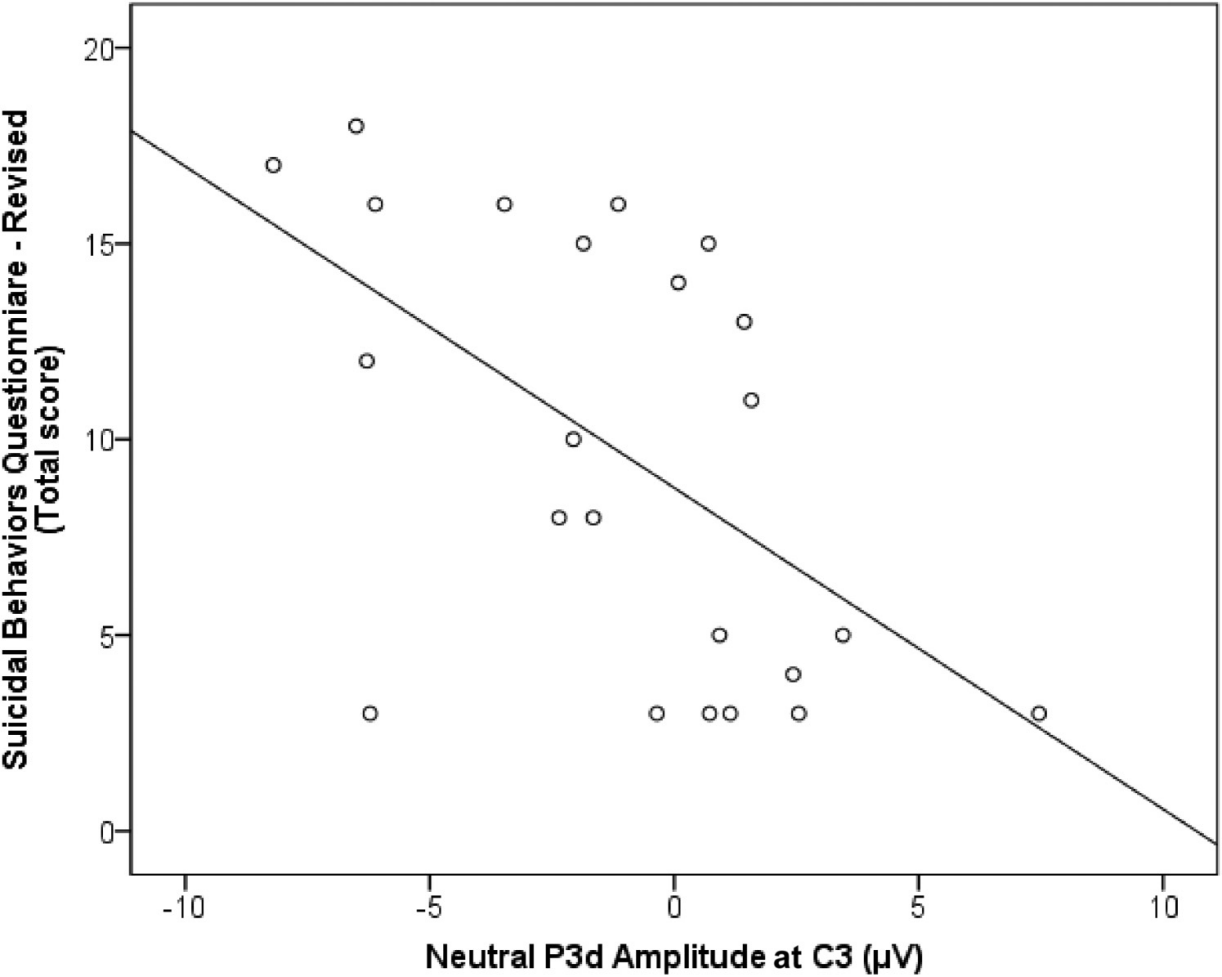
Association between suicidal symptoms and the neural response to inhibition. Large significant correlation between total scores on the Suicidal Behaviors Questionnaire-Revised (SBQ-R) and the P3d amplitude in response to emotionally neutral (*P* = .004).

There was no significant correlation between CDI-2 total scores and the P3d amplitudes. A negative correlation with a large effect size (*r*_s_ = −.57, *P* = .004) showed that higher suicidality on the SBQ-R was significantly associated with lower P3d amplitude evoked by emotionally neutral stimuli in C3 (Figure 5). Sensitivity analyses confirmed that this remained significant without the single participant from the suicidal group who was not using antidepressant medications (*r*_s_ = −.58, *P* = .005) and after controlling for other types of psychotropic medications (*r*_s_ = −.52, *P* = .020). There was no other significant correlation between the SBQ-R and the P3d amplitude for other emotional conditions, nor in C4.

## Discussion

Very few studies have investigated the neural correlates of acute suicide in psychiatric inpatients. The present study was carried out with adolescent inpatients, only days after being admitted to a psychiatric inpatient unit because of an acute risk of suicide. Findings show that while suicidal adolescents may perform similarly to healthy controls during an emotional Go/NoGo task (as indexed by behavioral data), underlying cognitive processes serving inhibitory control present significant abnormalities that are influenced by emotional valence and hemispheric asymmetries.

Similar RT and response accuracy were observed in suicidal adolescents compared to healthy controls. These results echo other behavioral findings for response inhibition tasks.^18–20,50–52^ In both groups, adolescents were faster to respond to happy faces, suggesting that they may more easily recognize positive emotional valence. This finding is similar to those from previous studies in adults.^42,53,54^ Additionally, both groups had lower accuracy when required to withhold a response to sad faces, suggesting that they had more difficulty to inhibit a response to negative stimuli. Importantly, these performance data do not suggest major differences in the ability to inhibit a response between the control and suicide groups. This is in marked contrast to some of the physiological data in which significant group differences were found.

No significant group differences or interactions involving groups were found for the N2d. Thus, there was no evidence of abnormal conflict monitoring, novelty detection or stimuli classification processing in the suicidal group. Previous studies have found larger N2d amplitudes in adults with a history of a suicide attempt compared to adults with suicidal ideation alone.^35^ The lack of effect on the N2d in the current study could possibly indicate that conflict detection processes may be less sensitive to suicidality in adolescents than in adults. This discrepancy may also reflect differences in other sample characteristics. Whether differences in the interactions between emotion processing and conflict detection may distinguish adolescents with suicidal behavior from those who do not act on their suicidal ideation remains to be investigated.

Conversely, significant group differences were found for indices of later inhibition processes. Compared to controls, the suicidal group exhibited reduced positivity at the time of the P3d in response to happy and neutral faces, while there was no significant group difference in response to sad faces. Further analyses of the underlying raw components revealed that these group differences resulted from lower P3 amplitudes following NoGo stimuli compared to Go stimuli for the suicidal group, while there was little change between Go and NoGo stimuli for controls. This suggests that suicidal adolescents may have difficulties recruiting neural resources to inhibit responses to neutral- and positively valenced information. By comparison, the processing of negative stimuli seemed to be preserved, as there was no significant group difference for sad faces. Hence, adolescent suicide attempters may be able to allocate more resources to process negatively valenced information, a type of information towards which they are known to have a bias.^55,56^ Furthermore, more severe suicidal symptoms were strongly associated with lower amplitude of the P3d in response to neutral faces, suggesting that some of the neural abnormalities underlying inhibitory processes may progressively worsen with the degree of suicidal behaviors.

Overall, the present findings are reminiscent of the previous observation of a reduced P3d amplitude in adults with depression relative to healthy controls during an emotionally neutral task.^49^ We further observed that this effect is modulated by emotional valence and hemispheric differences in suicidal adolescents. During the presentation of happy facial expressions, the group difference for the P3d was largest in the right hemisphere, revealing greater asymmetry in inhibition processing for these types of stimuli in the suicidal group. This finding is similar to those of previous fMRI studies using emotional Go/NoGo paradigms which reported reduced activations in the right hemisphere linked to inhibition in impulsive individuals.^57,58^ Overall, expanding on previous findings,^59^ the current findings suggest that there are interactions between inhibition, emotion processing and cerebral hemisphere, with potential implications for the understanding of cognitive-affective brain processing associated with adolescent suicidality.

This study has some limitations. The sample size was small. However, previous ERP studies with suicidal populations have used sample sizes similar to the present study,^60–64^ highlighting the reality of difficulties with participant recruitment in this vulnerable population and the potential for relevant clinical findings with a limited sample. Furthermore, since most participants in the suicidal group also had depression and were medicated, it was not possible to disentangle the potential effects of suicidality from that of mood disorders or medication. Nevertheless, mood disorders and related medications are highly common in adolescents with suicidal behavior and therefore, the results of the present study are likely to be representative of a large portion of that population.

The present study is the first to examine interactions between inhibitory control and emotion processing in acutely suicidal adolescents. The P3 data suggest that, compared to healthy adolescents, adolescents who recently attempted suicide may have fewer neural resources for inhibitory control, particularly for information with a positive emotional valence. These findings suggest that emotional context may influence inhibitory control difficulties during a suicidal crisis. Future studies with larger sample sizes should further investigate the distinct correlates of depression, suicidality, and medication on the interactions between emotional and executive processing.
